# Magnesium-Sodium Hybrid Battery With High Voltage, Capacity and Cyclability

**DOI:** 10.3389/fchem.2018.00611

**Published:** 2018-12-10

**Authors:** Ruigang Zhang, Oscar Tutusaus, Rana Mohtadi, Chen Ling

**Affiliations:** Materials Research Department, Toyota Research Institute of North America, Ann Arbor, MI, United States

**Keywords:** hybrid battery, Mg-Na battery, Mg, NaCrO_2_, post Li-ion battery, energy density

## Abstract

Rechargeable magnesium battery has been widely considered as a potential alternative to current Li-ion technology. However, the lack of appropriate cathode with high-energy density and good sustainability hinders the realization of competitive magnesium cells. Recently, a new concept of hybrid battery coupling metal magnesium anode with a cathode undergoing the electrochemical cycling of a secondary ion has received increased attention. Mg-Na hybrid battery, for example, utilizes the dendritic-free deposition of magnesium at the anode and fast Na^+^-intercalation at the cathode to reversibly store and harvest energy. In the current work, the principles that take the full advantage of metal Mg anode and Na-battery cathode to construct high-performance Mg-Na hybrid battery are described. By rationally applying such design principle, we constructed a Mg-NaCrO_2_ hybrid battery using metal Mg anode, NaCrO_2_ cathode and a mixture of all-phenyl complex (PhMgCl-AlCl_3_, Mg-APC) and sodium carba-*closo*-dodecaborate (NaCB_11_H_12_) as dual-salt electrolyte. The Mg-NaCrO_2_ cell delivered an energy density of 183 Wh kg^−1^ at the voltage of 2.3 V averaged in 50 cycles. We found that the amount of electrolyte can be reduced by using solid MgCl_2_ as additional magnesium reservoir while maintaining comparable electrochemical performance. A hypothetical MgCl_2_-NaCrO_2_ hybrid battery is therefore proposed with energy density estimated to be 215 Wh kg^−1^ and the output voltage over 2 V.

## Introduction

Since the first commercialization in 1991, lithium-ion battery (LIB) has become the dominating power source in the market of portable electronics. While expanding the territory of current Li-ion technology into larger scale devices faces challenges on the energy density, high materials cost, safety issues and potential supply risk, great efforts have been devoted to developing beyond Li-ion chemistries. One of such non-Li-based candidates is rechargeable magnesium battery (Yoo et al., 2013; Muldoon et al., 2014). Metallic magnesium anode has a volumetric capacity of 3,833 mAh cm^−3^ nearly doubling to that of lithium, 2,061 mAh cm^−3^. In addition, the deposition of magnesium forms non-dendritic morphology (Matsui, 2011; Ling et al., 2012), overcoming one of the major safety risks of dendritic lithium plating. Encouraged by these significant benefits, the research of magnesium battery has gained much attention and several breakthroughs have been made in the past few years (Yoo et al., 2013; Canepa et al., 2017). At this moment the biggest challenge toward the realization of a competitive magnesium battery is to marry metal magnesium anode with a cathode providing high energy density, rate capability and good cyclability (Yoo et al., 2013; Muldoon et al., 2014; Bucur et al., 2015). The bivalency of Mg^2+^ results in undesirable effects that prevent the insertion of magnesium in many host materials (Mizuno et al., 2014; Emly and Ven, 2015; Ling et al., 2015, 2016; Liu et al., 2015; Wan et al., 2015; Zhang and Ling, 2016; Ling and Suto, 2017). As a result, only a few cathodes including Chevrel phase Mo_6_S_8_ and spinel TiS_2_ have shown remarkable cyclability as Mg battery cathode (Aubach et al., 2000; Sun et al., 2016). The energy densities of sulfide-based cathodes are, however, limited by their voltages, typically lower than 1.5 V (Liu et al., 2016).

To avoid the technically difficult Mg^2+^-intercalation, recently a concept of hybrid battery was proposed, where a secondary type of cation replaces Mg^2+^ to participate in the cathodic reaction (Yao et al., 2016). In the operation of hybrid battery, the anode experiences the deposition and dissolution of metal magnesium, while the cathode undergoes a fast and reversible electrochemical reaction to balance the charge neutrality in the electrolyte. Compared to the shuttling of charge carrying ions between positive and negative electrodes in classical “rocking-chair” configuration, hybrid battery provides new opportunities to exhibit impressive properties by taking the synergy between the combinational use of electrodes and electrolyte. Good electrochemical performance such as high rate capability, cyclability, and Columbic efficiency was demonstrated in several Mg-Li hybrid cells (Cheng et al., 2014, 2016; Gao et al., 2015; Yoo et al., 2015; Fan et al., 2017). However, the use of Li-intercalation in the cathode reaction still shares the concerns of Li-ion based technology such as high-cost materials and potential supply risks.

Beyond Li-based battery systems, the study of Li-free hybrid battery has also attracted attention. Of particular interest is Mg-Na hybrid battery, aiming to take the full advantage of metal Mg anode (high capacity, availability, low cost, safety) and Na-battery cathode (high voltage and energy density, availability, low cost, cyclability). Walter et al. used metal Mg anode and FeS_2_ nanocrystal cathode and cycled the cell in a dual-salt electrolyte containing both Mg^2+^ and Na^+^ (Walter et al., 2015). The hybrid Mg-(Na_x_)FeS_2_ battery showed good cathodic capacity, high Columbic efficiency and rate capability (Walter et al., 2015). Yao and his coworkers demonstrated the first intercalation-type Mg-Na hybrid battery using the open framework Berlin green cathode (Dong et al., 2016). Their Mg-Berlin green hybrid cell delivered an average discharge voltage of 2.2 V and stable cycling for 50 cycles (Dong et al., 2016). By using a Na_3_V_2_(PO_4_)_3_ cathode, the voltage and energy density of Mg-Na hybrid battery was further increased to 2.6 V and 150 Wh kg^−1^, respectively, and good capacity retention at high rates was observed (Li et al., 2017).

While in the past few years many electrode materials were identified as the host for sodium intercalation (Yabbuchi et al., 2014), it is optimistically expected that the electrochemical performance of Mg-Na hybrid battery can someday be improved to reach the level of consideration in large scale applications (Li et al., 2017). In the current work, the principles to construct high-performance Mg-Na hybrid battery from the knowledge of Na-battery and Mg-battery research are detailed. These principles led us to rationally design a new Mg-Na hybrid battery using metal Mg anode, NaCrO_2_ as Na^+^-intercalation cathode and a mixture of all-phenyl complex (PhMgCl-AlCl_3_, Mg-APC) and sodium carba-*closo*-dodecaborate (NaCB_11_H_12_) as dual-salt electrolyte. The Mg-NaCrO_2_ hybrid cell delivered an energy density of 183 Wh kg^−1^ at the voltage of 2.3 V averaged in 50 cycles. In addition, while hybrid battery is usually prone to the reduction of energy density at cell level due to the necessary usage of sufficient electrolyte to store charge carrying ion, we found that the amount of electrolyte can be reduced by using solid MgCl_2_ as additional magnesium reservoir while maintaining comparable electrochemical performance. A hypothetical MgCl_2_-NaCrO_2_ hybrid battery is therefore proposed with energy density estimated to be 215 Wh kg^−1^ and the output voltage over 2 V.

## Experiment

### Material Synthesis and Characterization

The synthesis of carbon coated NaCrO_2_ was carried out via a solid-state reaction route. The starting materials of sodium oxalate (Na_2_C_2_O_4_, ≥99.5%, Sigma-Aldrich), chromium(III) oxide (Cr_2_O_3_, 99.9%, Sigma-Aldrich) and glucose were mixed in a molar ratio of 1:0.5:0.15 and ball milled in a zirconia pot at 500 rpm for 1 h. The mixture was heated at 400°C for 2 h in argon. The obtained powder was then pressed to pellet and calcined at 900°C for 10 h in argon flow. Sodium carba-*closo*-dodecaborate (NaCB_11_H_12_) was obtained by first preparing an aqueous solution of (H_3_O)[CB_11_H_12_] from CsCB_11_H_12_ (5.13 g, 18.5 mmol) using an ion exchange column (Dowex 50WX8 50–100 mesh, 50 mL), followed by neutralization with 0.1 M NaOH until pH 7. The aqueous solution containing NaCB_11_H_12_ was evaporated to dryness and the solid was extracted with anhydrous Et_2_O (20 mL) discarding any insoluble solid; then the solvent was removed under reduced pressure and the resulting oil was further dried under vacuum at 100°C overnight to yield 2.98 g (96%) of anhydrous NaCB_11_H_12_. All other Na electrolytes (Solvionic, 99.9%, H_2_O content ≤ 20 ppm) were used as received without additional purification. Mg-APC electrolyte was prepared by dissolving aluminum trichloride (AlCl_3_, anhydrous, 99.99%, Sigma-Aldrich) in tetrahydrofuran (THF, anhydrous, >99.9%, Sigma-Aldrich) and mixing the solution with phenyl magnesium chloride (PhMgCl, 2M solution in THF, Sigma-Aldrich). X-ray diffraction measurements were performed at room temperature on a Rigaku SmartLab diffractometer using Cu K_α_ radiation. The diffraction data were collected in a 2θ range of 10–70° with a step size of 0.05° and a scan rate of 0.04°/min. Morphology images were taken with a JOEL 7900 field emission scanning electronic microscope (SEM) and Hitachi HR-9000 transmission electron microscope (TEM).

### Electrochemical Measurement

The performance of Mg-Na hybrid cell was tested in a customized Tomcell (TJ-AC Tomcell Japan) using a 0.2 mm thick (28 mm diameter) standard glass filter (Sigma-Aldrich) as a separator and a Mg foil (19 mm diameter) as the counter and reference electrodes. Mg foil was polished by scraping each side of the foil with sandpaper and wiping clean with a Kimwipe (Kimberly-Clark). The NaCrO_2_ electrodes were composed of 80 wt% active material, 10 wt % Ketjen black and 10 wt% polytetrafluoroethylene binder. The cycling was performed in the voltage region between 0.5 and 3.2 V vs. metal Mg using a Bio-Logic potentiostat/galvanostat VMP battery testing system.

### Computational Method

The theoretical study was based on density functional theory (DFT) calculations performed with the Vienna ab initio Simulation Package (VASP) using projector-augmented waves (PAW) pseudopotentials and the exchange-correlation functionals parametrized by Perdew, Burke, and Ernzerhof for the generalized gradient approximation (GGA) (Kresse and Hafner, 1994; Kresse and Furthmuller, 1996; Kresse and Joubert, 1999). Numerical convergence to about 2 meV per formula unit was ensured by using a cutoff energy 520.0 eV and appropriate Gamma centered k-point meshes. The energy barriers for the migration of Mg and Na were calculated with the climbing-image nudged elastic band (cNEB) method in 4 × 4 × 1 supercells.

## Results

### Design Cell Configuration

**Figure 1:**
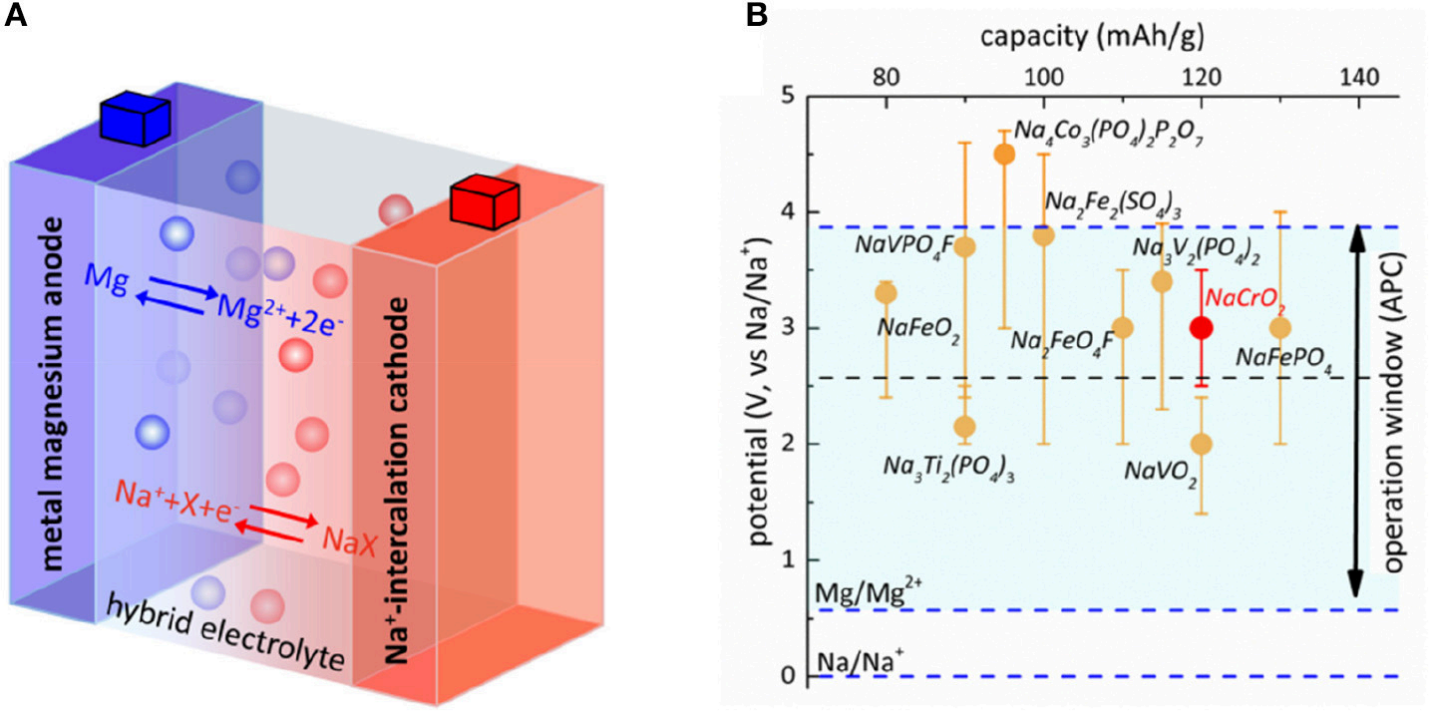
Design of Mg-Na hybrid battery. **(A)** Illustration of working principle of Mg-Na hybrid cell. **(B)** Align the window of stability of Mg-APC (blue dashed line) and Mg(BH_4_)_2_ (black dashed line) and the operating range of voltage for several Na-battery cathodes.

As illustrated in Figure 1A, the Mg-Na battery desirably works via Na^+^-insertion/extraction at the cathode and Mg dissolution/deposition at the anode in the discharge/charge cycling. A rule of thumb to construct a battery configuration is that the battery should be operated in the range to avoid undesirable side reactions that deteriorate cycling capability. In particular, the cell cannot be charged to voltage too high to cause the spontaneous oxidation of electrolyte while the discharge voltage cannot be too low to reduce electrolyte (Goodenough and Kim, 2010). Hence, the window of stability for the oxidation and reduction of electrolyte must match the operating voltages of electrodes. This gives the first principle to select appropriate configuration of Mg-Na hybrid battery. Applying the oxidation stability of electrolytes in Mg half-cell plus the potential difference for Mg and Na deposition, the window of stability can be aligned with the reported operating voltage of cathodes in Na half-cells. Note here we should consider the full range of operating voltages of electrode instead of the average voltage of charge/discharge. In Figure 1B, we plot the oxidative stability for the two well-known Mg electrolytes Mg(BH_4_)_2_ and Mg-APC, 2.0 V and 3.3 V vs. metal Mg, respectively (Mohtadi et al., 2012), in comparison with the operating range of several Na-battery cathodes (Yabbuchi et al., 2014). The potential difference for Mg and Na deposition in tetrahydrofuran (THF) solution was 0.57 V as measured from cyclic voltammetry (Figure S1). Clearly, the low oxidation stability of Mg(BH_4_)_2_ restricts the choice of cathode to Ti^3+^/Ti^4+^ (Na_3_Ti_2_(PO_4_)_3_) and V^3+^/V^4+^ (NaVO_2_) redox couples and most other intercalation-type cathodes work in the voltage higher than the allowed window of stability. Because of the higher oxidation stability of Mg-APC electrolyte, the usage of redox couples of higher voltages such as Fe^2+^/Fe^3+^, V^4+^/V^5+^, and Cr^3+^/Cr^4+^ becomes possible. Of particular interest in Figure 1B is NaCrO_2_ cathode. In sodium battery, approximately 50% of sodium ions can be reversibly extracted from NaCrO_2_ at a voltage plateau of nearly 3.0–3.3 V vs. Na metal. Further extraction of sodium ions beyond 50% results in complex phase transitions, leading to the loss of reversibility (Kubota et al., 2015; Bo et al., 2016). The reversible capacity of NaCrO_2_ is ~120 mAh/g and the operation range well matches the stability window of Mg-APC electrolyte, making it a suitable cathode for Mg-Na hybrid battery.

After selecting the cathode, the next step is to find an electrolyte for the hybrid cell. Because the Mg-NaCrO_2_ cell is a discharged configuration, the electrochemical operation starts with the charge process extracting Na^+^ from the cathode and depositing Mg on the anode. The capacity of the cell is limited if the electrolyte cannot provide sufficient Mg^2+^ ions to balance the cathodic reaction. On the other side, pure Mg electrolyte also hinders the electrochemical reaction probably due to the lack of Na^+^ for cathodic reaction. Based on these considerations, the electrolyte should store Mg^2+^ and support fast Na^+^ conduction in addition to the conventional function of charge transfer. To find an appropriate candidate, we mixed Mg-APC with one of Na-electrolytes, NaPF_6_, NaClO_4_, and Na(CB_11_H_12_) and conducted galvanostatic tests to examine the electrochemical capability of these dual-salt electrolytes. In Na/NaCrO_2_ half-cells, all three dual-salt electrolytes performed similarly well and the voltage profiles were comparable to that using pure Na-electrolyte (Figure S2), indicating that adding Mg-APC in Na half cells did not display any appreciable influence of electrochemical activity. In Mg/Mg symmetric cells, stable Mg deposition and dissolution was only observed using Mg-APC/Na(CB_11_H_12_) electrolyte, while cells containing NaClO_4_ or NaPF_6_ were not electrochemically active (Supporting Information). Consistently, in the full cell test of Mg/NaCrO_2_, batteries using Mg-APC/NaClO_4_ or Mg-APC/NaPF_6_ had negligible capacity (< 1 mAh g^−1^). Though we cannot completely rule out the possibility that the trace amount of impurities contained in the Na electrolytes such as moisture in a few tens of ppm level severely affect the operation of the full cell, our results are consistent with the previous work that PF${}_{6}^{-}$ anions passivate Mg anodes and completely inhibit any Mg deposition/dissolution process (Shterenberg et al., 2017). Clearly, the choice of electrolyte is crucial to support the anodic reaction in Mg-Na hybrid battery. Both Mg-APC and Mg(CB_11_H_12_)_2_ are active electrolytes for Mg deposition/dissolution;(Tutusaus et al., 2015) hence adding Na(CB_11_H_12_) into Mg-APC does not generate any inactive species for anodic reaction. In contrast, the incompatibility of ClO${}_{4}^{-}$ and PF${}_{6}^{-}$ anions with magnesium metal anode, prohibits anodic reaction in electrolyte solutions containing NaClO_4_ or NaPF_6_, resulting in poor electrochemical performance (Singh et al., 2013; Yoo et al., 2013) Interestingly, adding NaPF_6_ or NaClO_4_ slightly also decreased the oxidation stability of Mg-APC as shown by a charge plateau responsible for the electrolyte decomposition at 3.18 and 3.12 V vs. Mg metal, respectively, which is probably due to the trace impurities from Na electrolytes and should be a subject for future research. For comparison, the Mg-APC/Na(CB_11_H_12_) electrolyte did not show any appreciable decomposition below 3.3 V vs. Mg metal.

The dual-salt electrolyte of Mg-APC and Na(CB_11_H_12_) is not simply a mixture of two solutions. Instead, the interaction between various active ions provides a synergy to improve the electrochemical performance of Mg-Na battery. Due to the low solubility, the introduction of Na^+^ into Mg-APC induces the precipitation of NaCl (Figure S4). Although the detailed analysis of the reaction mechanism is beyond the scope of current work and should be considered in future research, a possible reaction is

(1) $${\left[{\text{Mg}}_{\text{2}}{\text{Cl}}_{\text{3}}\right]}^{+}+{\text{Na}}^{+}\to \text{2}{\left[\text{MgCl}\right]}^{+}+\text{NaCl}$$

It therefore shifts the equilibrium toward the formation of [MgCl]^+^, arguably the electro-active carrier for Mg^2+^ deposition (Kim et al., 2014; Liu et al., 2014) providing an effective way to tune the performance of the Mg-NaCrO_2_ battery by controlling the ratio between Mg-APC and Na(CB_11_H_12_) in dual-salt electrolyte. Using non-carbon-coated NaCrO_2_ cathode, we found that the highest discharge capacity was achieved when using a 3:7 volumetric mixture of 0.4 M Mg-APC and 0.5 M Na(CB_11_H_12_), which was the dual salt electrolyte used in the following tests.

These results are summarized in the following principles to construct high-performance Mg-Na hybrid battery:

1. The operating voltage range of Na-cathode should not exceed the stability of Mg-electrolyte. Especially, the charge of Na-cathode should not exceed the oxidation stability of Mg-electrolyte.
2. For a cell starting from discharged configuration, the electrolyte should contain a mixture of Mg^2+^ and Na^+^ and should not include any species that prevents the reversible deposition/dissolution of magnesium.
3. The composition of dual-salt electrolyte should be tuned to support the transport of both Mg^2+^ and Na^+^ while keeping sufficient supply of Mg^2+^ if the battery is assembled at discharged state and Na^+^ if the battery is assembled at charged state.

### Electrochemical Performance and Reaction Mechanism

**Figure 2:**
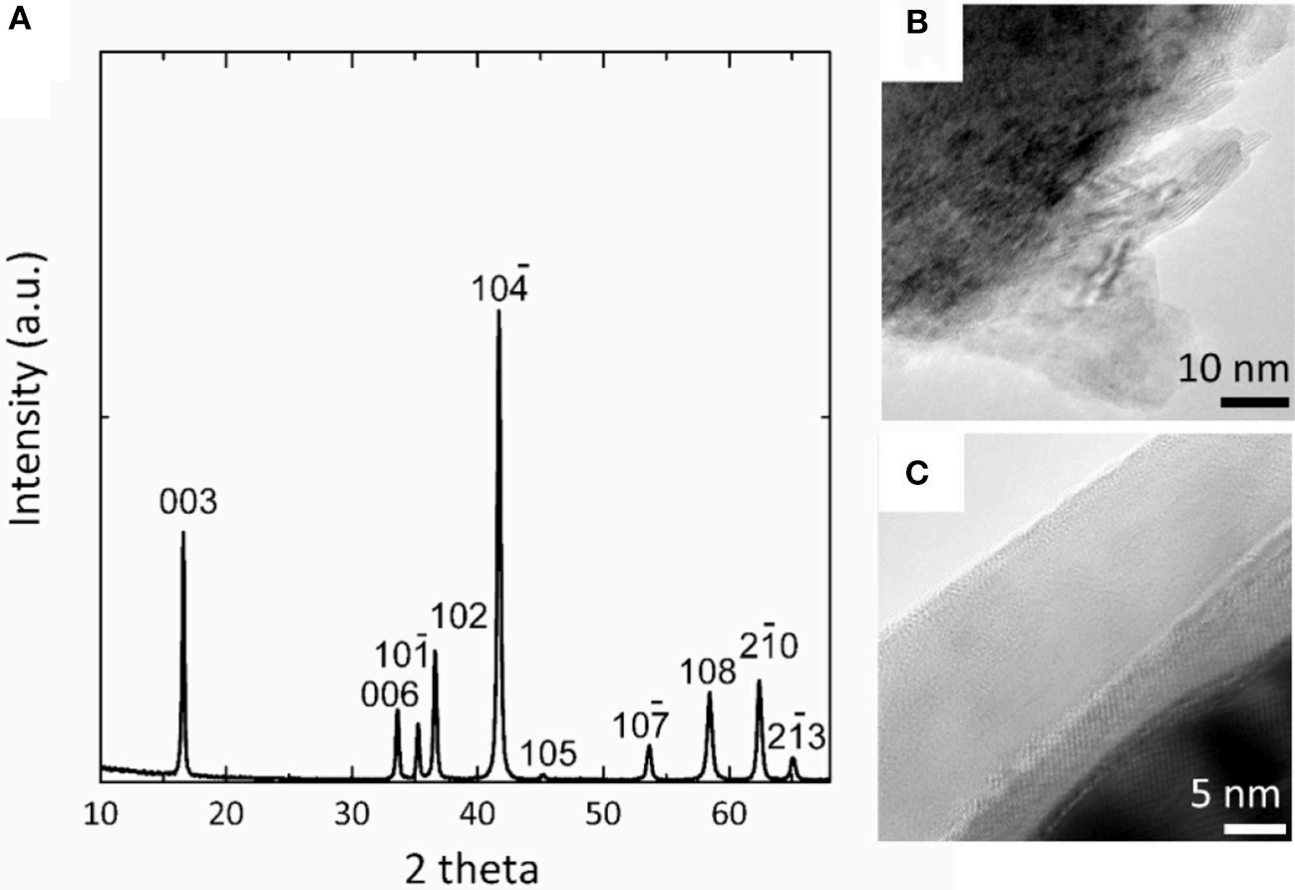
Characterization of synthesized NaCrO_2_. **(A)** X-ray diffraction of NaCrO_2_. **(B)** TEM of uncoated NaCrO_2_ particles. **(C)** TEM of a NaCrO_2_ particle with carbon coating.

By rationally applying the above principles, we assembled a Mg-Na hybrid battery using metal Mg anode, NaCrO_2_ cathode and a dual-salt electrolyte prepared by mixing 0.4 M Mg-APC and 0.5 M Na(CB_11_H_12_) in 3:7 volumetric ratio. To protect the surface of NaCrO_2_ from the formation of carbonate and to enhance the electronic conductivity of cathode, NaCrO_2_ was coated with carbon. The X-ray diffraction pattern matched excellently to the rhombohedral structure of NaCrO_2_ (space group: R3) without any appreciable peak of carbon (Figure 2A). The synthesized NaCrO_2_ showed micron-sized particles with plate-like structures. TEM images of uncoated NaCrO_2_ exhibited high crystallinity and smoothly developed edges on surface (Figure 2B). In the coated sample, a uniform carbon layer was clearly observed, as shown in Figure 2C. The thickness of carbon layer was estimated to be about 15 nm.

**Figure 3:**
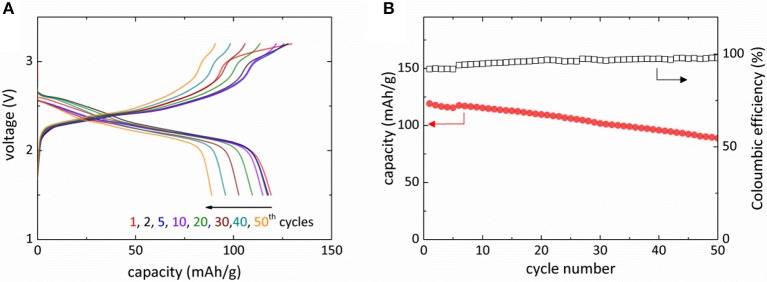
Electrochemical performance of Mg-NaCrO_2_ hybrid battery. **(A)** Voltage profile for the charge and discharge of Mg-NaCrO_2_ hybrid battery. **(B)** Discharge capacity and coulombic efficiency during the first 50 cycles.

Figure 3A presents the galvanostatic charge-discharge curves of Mg-NaCrO_2_ cell. The electrochemical cycling was operated in the voltage range of 1.5–3.2 V and a rate of 0.05 C (1 C = 120 mAh g^−1^). A 3.2 V charging cutoff voltage was selected to avoid electrolyte decomposition and to alleviate the irreversible phase transition in the cathode material caused by the extraction of more than 0.5 Na^+^ (Komaba et al., 2009; Kubota et al., 2015). The battery started with an open-circuit voltage of 1.68 V and the charge increased the voltage to a plateau at approximately 2.4 V. An additional plateau at about 3.0 V appeared before the charge was terminated at 3.2 V. The discharge of Mg-NaCrO_2_ battery was highly reversible with the initial discharge capacity reaching 118 mAh g^−1^ and an average capacity of 105 mAh g^−1^ during the first 50 cycles (Figure 3B). Note here the capacity was evaluated based on the weight of active cathode material. The average discharge voltage was 2.32 V, ~0.7 V lower than that measured in Na-ion cell. The difference was attributed mainly to the different anodic potential between Mg/Mg^2+^ and Na/Na^+^ (~0.57 V), albeit the change of cation chemical potential may also contribute to the shift of voltage profile. The appropriate choice of cell configuration resulted in the high coulombic efficiency of Mg-NaCrO_2_ cell, 91% in the first cycle and gradually stabilizing at 98% after 25th cycle (Figure 3B).

**Figure 4:**
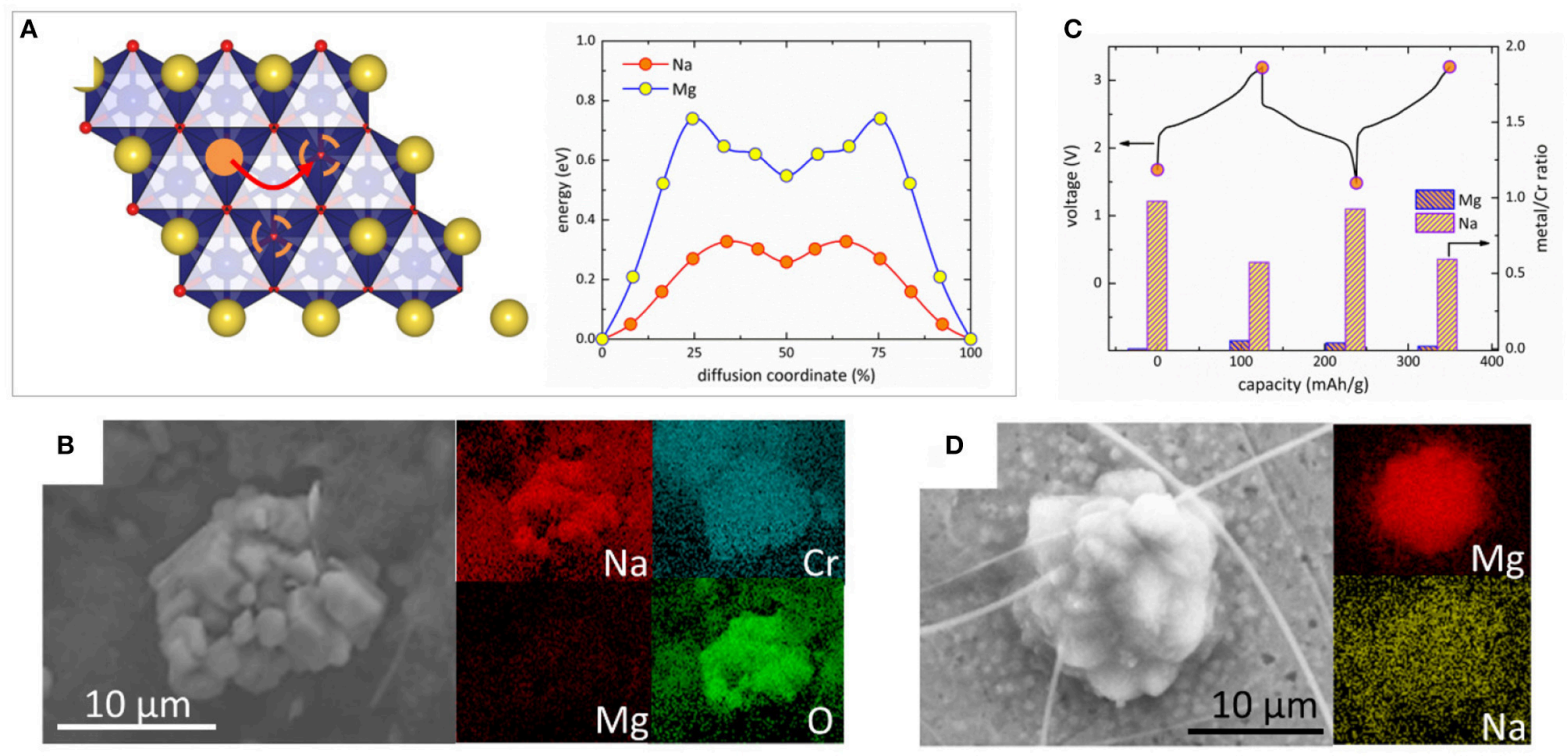
Analysis of the electrochemical reactions at the NaCrO_2_ cathode and metal Mg anode in Mg-NaCrO_2_ cell. **(A)** Diffusion barrier of Mg^2+^ and Na^+^ in the divacancy mechanism. The mobile ion is shown as solid orange circle while the vacancies are shown as dashed circles. Na and CrO_6_ octahedra are shown in yellow and blue color, respectively. **(B)** SEM and EDS analysis of discharged cathode. **(C)** Variation of Mg and Na content in the cathode measured by EDS in two charge-discharge cycles. **(D)** SEM and EDS analysis of a particle deposited on stainless steel anode in charge. The spikes are ceramic residuals from the separator.

In hybrid Mg-Na batteries, the largely different charge and ionic size makes the co-insertion of Mg^2+^ and Na^+^ very unlikely, in contrast to the co-insertion of Mg^2+^ and Li^+^ in some hybrid Mg-Li cells (Cho et al., 2014; Wu et al., 2015). In addition, the lack of eutectic region in the binary Mg-Na phase diagram below the melting point of Na (371 K) indicates that the anodic Mg deposition/dissolution without Na-alloying can be expected. Thus, the hybrid Mg-Na system has potential benefit to control the reversible electrochemical reactions compared to the hybrid Mg-Li cells. We carried out several analyses to determine the reaction mechanism in Mg-NaCrO_2_ hybrid battery. The cathodic reaction could in principle occur through two primary paths, the extraction/insertion of sodium ions or of magnesium ions. Density functional theory (DFT) calculations were performed to assess the mobility of Mg^2+^ and Na^+^ in the lattice of Na_x_CrO_2_. Previous work showed that the diffusion of alkali ions in O3 layered oxides is dominated by the divacancy hopping (Ven et al., 2001), where the mobile ion in octahedral site migrates through the intermediate tetrahedral site surrounded by two vacancies (Figure 4A). In the divacancy mechanism, the migration barrier of Mg^2+^ is 440 meV higher than that of Na^+^. Considering that a practical intercalation generally requires the diffusion barrier of mobile ion less than ~525 meV (Liu et al., 2015), the insertion of Mg^2+^ in Na_x_CrO_2_ is kinetically difficult while the mobility of Na^+^ is sufficient for intercalation at a reasonable rate. This conclusion was confirmed in the control experiment using metallic Mg anode, partially desodiated NaCrO_2_ as the cathode (x ≈ 0.5) and pure Mg-APC electrolyte. After creating sufficient number of empty sites between CrO_2_ layers by desodiation, negligible capacity (< 4 mAh g^−1^) responsible for Mg insertion was recorded in a discharge experiment (Figure S3), denying the possibility of Mg^2+^-intercalation in Na_x_CrO_2_.

The elemental composition of the cathode at different electrochemical stages was probed by *ex situ* energy-dispersive X-ray spectroscopy (EDXS). A SEM image of a typical cathode particle after 1st charge-discharge cycle is shown in Figure 4B. The EDXS mapping showed a uniform spatial distribution of Na over the particle, while very weak Mg signal was observed probably only due to the residue of electrolyte adsorbed on the surface. Figure 4C showed the variation of Na and Mg content in the cathode in two electrochemical cycles. While little amount of Mg was detected, the change of Na concentration quantitatively agreed with the electrochemically measured capacity. These results demonstrated that the cathode reaction of Mg-NaCrO_2_ battery only involved Na^+^ extraction and insertion despite the existence of Mg^2+^ in the dual-salt electrolyte.

To study the anodic reaction, we replaced Mg metal anode with stainless steel (SS) to avoid the background EDXS signal from Mg foil. After charge, we observed the aggregation of nano-sized sphere-like primary particles into some micro-sized round particles on the SS surface. Figure 4D shows the SEM image of one of such particles. The elemental composition of the deposit showed the particle was primarily composed of Mg without any appreciable level of Na or other elements in the electrolyte, such as Al, Cl, B, and C. Although the potential of Al deposition is higher than that of Mg, the anion aluminate species present the dual-salt electrolyte did not participate in the deposition process and the anodic reaction occurred exclusively via Mg deposition/dissolution.

Combining the results for cathodic and anodic reaction, we concluded that the electrochemical reaction of our Mg-NaCrO_2_ battery was the insertion/extraction of Na^+^ at NaCrO_2_ cathode and Mg dissolution/deposition at metallic Mg anode where the charge balance in the electrolyte is accomplished by exchanging Mg^2+^ and Na^+^

(2) $$\begin{array}{l} \text{Cathode}:2NaCrO_{2}⇄2Na_{0.5}CrO_{2}+Na^{+}+e^{-} \end{array}$$

(3) $$\begin{array}{l} \text{Anode}:Mg+Cl^{-}⇄MgCl^{+}+2e^{-} \end{array}$$

(4) $$\begin{array}{l} \text{Overall}:4NaCrO_{2}+MgCl^{+}⇄2Na^{+}+Cl^{-}\\ +\;4Na_{0.5}CrO_{2}+0.5Mg \end{array}$$

Assuming the overall reaction 4, the energy density of Mg-NaCrO_2_ calculated based on the weight of NaCrO_2_ and MgCl^+^ was 212 Wh kg^−1^ with average voltage of 2.3 V. Taken the full weight of Mg salt (PhMgCl) into consideration, the average energy density is 183 Wh kg^−1^, higher than the Mg-Na_3_V_2_(PO_4_)_3_ hybrid battery (150 Wh kg^−1^, 2.6 V) and the Mg-Berlin Green hybrid battery (135 Wh kg^−1^, 2.1 V).

**Figure 5:**
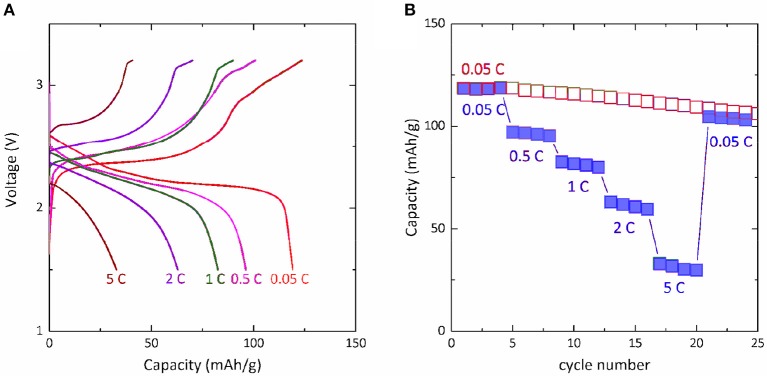
Rate performance of Mg-NaCrO_2_ hybrid cell. **(A)** Charge-discharge profile at different C rates. **(B)** Capacity retention at high rate cycling.

One important advantage of Mg-Na hybrid battery is that it avoids the kinetically sluggish Mg^2+^-insertion/extraction process, thus creating potential to achieve high rate capability. The high-rate performance of Mg-NaCrO_2_ cell was examined at 0.05, 0.5, 1, 2, and 5 C rate for four cycles each. At these higher rates, the cell delivered an average capacity of 96, 81, 61, and 31 mAh g^−1^, respectively (Figure 5). After the current was decreased back to 0.05 C rate, the cell retained the performance, implying no appreciable degradation during the high-rate operations. Because the anodic deposition and dissolution of Mg in Mg-APC electrolyte is a fast electrochemical process (Matsui, 2011), we believe that the high-rate capability of Mg-NaCrO_2_ is determined by the performance of NaCrO_2_ cathode. The cathode used in our test was prepared without any attempt to optimize its rate capability. Recent work showed that the high-rate performance of NaCrO_2_ cathode in Na cells can be greatly enhanced by controlling the synthesis and surface modification. For instance, through an emulsion-drying synthesis and carbon coating, Yu et al. was able to cycle NaCrO_2_ at ultrafast rate up to 150 C (Yu et al., 2015). We anticipate that the optimization of NaCrO_2_ cathode for high rate capability can certainly result in excellent rate performance in Mg-NaCrO_2_ hybrid cell, which is the subject of future research work.

### Use Solid MgCl_2_ as Magnesium Reservoir

**Figure 6:**
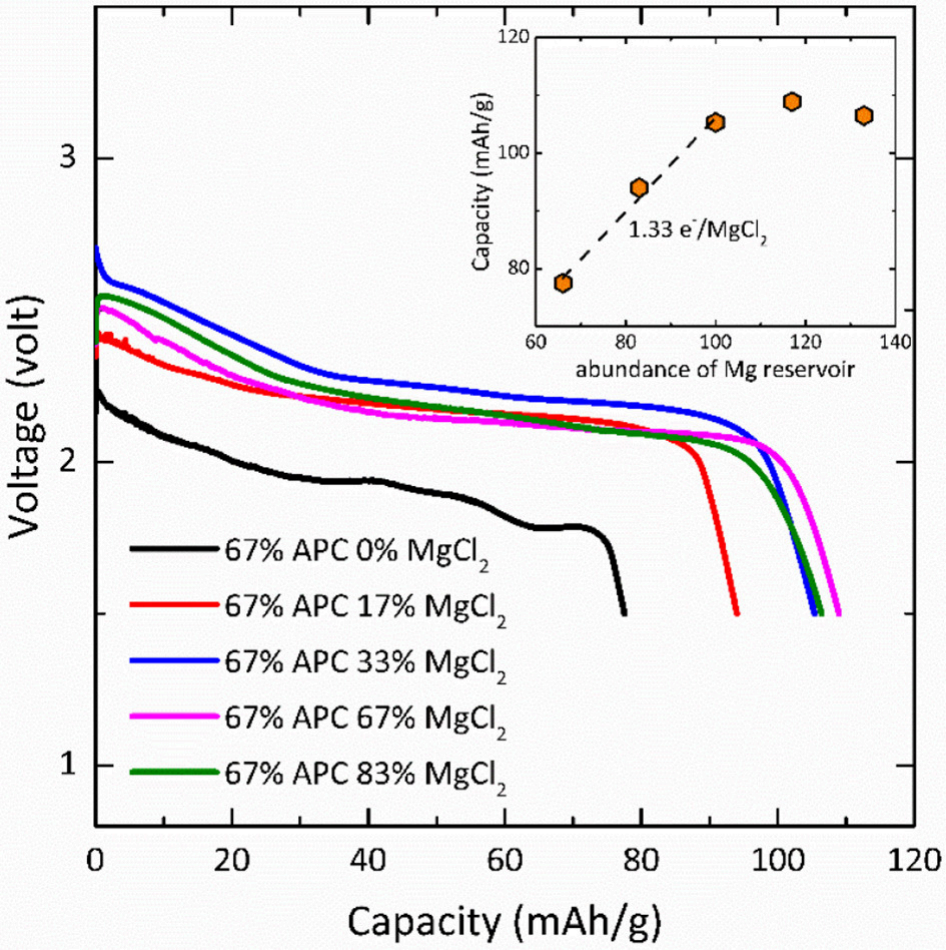
Discharge of Mg-NaCrO_2_ hybrid cell using solid MgCl_2_ as additional Mg reservoir. The percentage shows the ratio of cathode that can be fully reacted with the supply of Mg^2+^ in the electrolyte and in the MgCl_2_. Insert: discharge capacity as a function of Mg^2+^ supply.

In Li-ion battery, the primary function of electrolyte is to conduct Li^+^ ions between positive and negative electrodes. In a Mg-Na hybrid battery, the electrolyte not only transport Mg^2+^ and Na^+^ but also is involved in the reaction (Equation 4) and changes the composition during cycling. Strictly speaking, the evaluation of energy density of a hybrid battery should consider the amount of both electrode and electrolyte used in the cell (Dong et al., 2016; Li et al., 2017). As a consequence, the highest achievable capacity of the Mg/NaCrO_2_ hybrid battery reported above, purely utilizing liquid electrolyte as Mg^2+^ source, is restricted by the solubility of the magnesium salt in solution. One avenue to improve the cell energy density is to reduce the amount of solvent while keeping sufficient supply of Mg^2+^. The amount of electrolyte can be reduced by employing a Mg salt with reversible precipitation and dissolution as an additional solid Mg reservoir, capable of releasing Mg^2+^ into the electrolyte during charge and storing Mg^2+^ during discharge. To demonstrate the feasibility of using a solid magnesium salt as a highly concentrated Mg source, we reduced the amount of electrolyte to balance only 67% of charge transfer for theoretical Na extraction (half Na per NaCrO_2_) and supplied solid MgCl_2_ to the cell, yielding an electrolyte mixture with a formal Mg concentration of 24.4 M. Figure 6 shows the discharge profile of Mg-NaCrO_2_ cells using reduced amount of Mg-APC/Na(CB_11_C_12_) electrolyte with solid MgCl_2_ addition. When the total amount of Mg^2+^ in the cell was not sufficient to balance the cathode reaction, the capacity was reduced according to the available Mg content. Adding MgCl_2_ increased the capacity until reaching the critical point where the total amount of Mg^2+^ became sufficient for the cathode reaction. The highest capacity was recorded when a slight excess of Mg was used (109 mAh g^−1^), very close to the one obtained using excess liquid electrolyte (100 mAh g^−1^). Although we did not perform any optimization to achieve better capacity, the current results clearly suggested the feasibility of reducing the amount of dual-salt electrolyte by addition of an insoluble solid magnesium salt while maintaining comparable electrochemical performance. Linearly fitting the capacity with MgCl_2_ content showed that each MgCl_2_ provided 1.33 e^−^ for the electrochemical capacity, or in other words the efficiency of utilizing solid MgCl_2_ for Mg cycling was 67%. Interestingly, the discharge voltage also showed dependence on the amount of MgCl_2_ addition, which reflected the influence of complex equilibriums in the electrolyte on the cell voltage.

In the best scenario, we may assume that the majority, if not all, Mg^2+^ is stored in the form of a solid magnesium salt such as MgCl_2_. In addition to the electrochemical reaction (2) and (3), the battery operation involves the dissolution/precipitation of MgCl_2_

$$\begin{array}{l} \text{Electrolyte}:MgCl_{2}\left(solid\right)⇄Mg^{2+}\left(solution\right)+2Cl^{-} \end{array}$$

The full cell can be described according to

$$\begin{array}{lll} \text{Overall}:2NaCrO_{2} & + & 0.5MgCl_{2}\left(solid\right)⇄Na^{+}+2Na_{0.5}CrO_{2}\\ +\;Cl^{-}+0.5Mg \end{array}$$

In fact, Equation (6) describes the reaction of a Mg-Na hybrid battery using NaCrO_2_ cathode and MgCl_2_ anode. In our previous work, we showed that some metal chlorides functioned as active electrodes that release chlorine ion in the electrolyte in the operation (Zhang et al., 2015). In the hypothetical NaCrO_2_-MgCl_2_ battery, the electrolyte serves as a media for charge balance but not for the storage of Mg^2+^. Hence it overcomes the risk of reduced energy density due to the necessary usage of large amount of electrolyte.

The voltage of the hypothetical MgCl_2_-NaCrO_2_ battery is

$$\begin{array}{l} V≃2.1\;\left(volt\right){-}^{\Delta G}dis,NaCl{/}_{2e} \end{array}$$

where the first term comes from the free energy change for reaction 2*NaCrO*_2_+*xMgCl*_2_(*solid*)⇄2*xNaCl*(*solid*)+2*Na*_1−*x*_*CrO*_2_+*Mg* and the Δ*G*_*dis, NaCl*_ is the dissolution free energy of NaCl. Ignoring the contribution of the second term, the energy density of the hypothetical NaCrO_2_-MgCl_2_ battery is estimated as 215 Wh kg^−1^. With this level of energy density and an output voltage over 2 V, it is of great interest to examine the feasibility of using magnesium salt instead of metal magnesium as anode in hybrid Mg-Na battery.

## Conclusion

In conclusion, we show that the following principles are critical to construct appropriate configuration for Mg-Na hybrid battery:

1. The operating voltage range of Na-cathode should not exceed the stability of Mg-electrolyte. Especially, the charge of Na-cathode should not exceed the oxidation stability of Mg-electrolyte. The state-of-art Mg-electrolyte typically has the oxidative stability not higher than 3.5 V vs. metal magnesium currently. This principle disables the usage of Na-cathode with average voltages higher than 4 V unless future development of Mg-electrolyte could lift the oxidation stability to higher levels.
2. For a cell starting from discharged configuration, the electrolyte should be a mixture solution of Mg and Na salt and should not include any species that is not compatible with metal magnesium anode; otherwise reversible deposition/dissolution of Mg is blocked.
3. The composition of dual-salt electrolyte should be tuned to support the transport of both Mg^2+^ and Na^+^ while keeping sufficient supply of Mg^2+^ if the battery is assembled at discharged state and Na^+^ if the battery is assembled at charged state.

By applying these principles, we rationally selected metal Mg anode, NaCrO_2_ as Na^+^-intercalation cathode and a mixture of all-phenyl complex (PhMgCl-AlCl_3_, Mg-APC) and sodium carba-*closo*-dodecaborate (NaCB_11_H_12_) as dual-salt electrolyte. The Mg-NaCrO_2_ hybrid battery delivering an energy density of 183 Wh kg^−1^ at average voltage of 2.3 V. We demonstrated that the Mg-NaCrO_2_ cell operated via Na-intercalation at the cathode and Mg deposition/dissolution at the anode. By avoiding the kinetic sluggish Mg-intercalation, the Mg-NaCrO_2_ cell showed good capacity retention at high current densities. Furthermore, we showed that adding solid MgCl_2_ as additional magnesium reservoir reduced the necessary amount of electrolyte while maintaining comparable electrochemical performance. A MgCl_2_-NaCrO_2_ hybrid battery is hypothesized to avoid the reduction of energy density at the cell level due to the necessary amount of electrolyte to store Mg^2+^ ion. The energy density of the hypothetical battery is estimated to be 215 Wh kg^−1^ and the output voltage is over 2 V. Therefore, the hypothetical MgCl_2_-NaCrO_2_ hybrid battery provides a potential candidate for large scale applications if achievable.

## Author Contributions

CL conceived the idea, designed the project with RZ, performed the calculation and wrote the draft. RZ synthesized NaCrO_2_ and carried out the electrochemical experiments. OT and RM prepared Na(CB_11_H_12_). All authors participated in analyzing the results and finalizing the manuscript.

### Conflict of Interest Statement

The authors declare that the research was conducted in the absence of any commercial or financial relationships that could be construed as a potential conflict of interest.
