# Supplementary material for: Magnesium-Sodium Hybrid Battery With High Voltage, Capacity and Cyclability
Source: Front Chem. 2018 Dec 10;6:611. doi: 10.3389/fchem.2018.00611 (PMC6295519; doi:10.3389/fchem.2018.00611)
Supplement: Supplementary file 1 [file Data_Sheet_1.docx]

**Supporting Information**

for

**Magnesium-Sodium Hybrid Battery with High Voltage, Capacity and Cyclability**

Ruigang Zhang, Oscar Tutusaus, Rana Mohtanti and Chen Ling*

Toyota Research Institute of North America, 1555 Woodridge Avenue, Ann Arbor, Michigan, United States, 48105
e-mail: chen.ling@toyota.com

**S1.** Determine the difference between Mg and Na deposition potential

In aqueous solution, the difference between the standard redox potential of Na/Na^+^ (-2.71 V vs SHE) and Mg/Mg^2+^ (-2.372 V vs SHE) is 0.338 V. However, because the Mg-Na hybrid cell is operated in aprotic electrolyte solutions, the difference between Mg and Na deposition potential should be re-evaluated.





**Figure S1.** CV profile of Mg and Na deposition/dissolution in THF solutions. The measurement is performed using metal Mg as the working electrode. Note we use the difference of nucleation potential (marked as dashed lines) to estimate the difference of Mg/Mg^2+^ and Na/Na^+^ in Figure 1b. Another way is to use the zero-current crossing potential during the return sweep between metal deposition and dissolution, which will shift the shadowed operation window of APC by ~0.1 V.


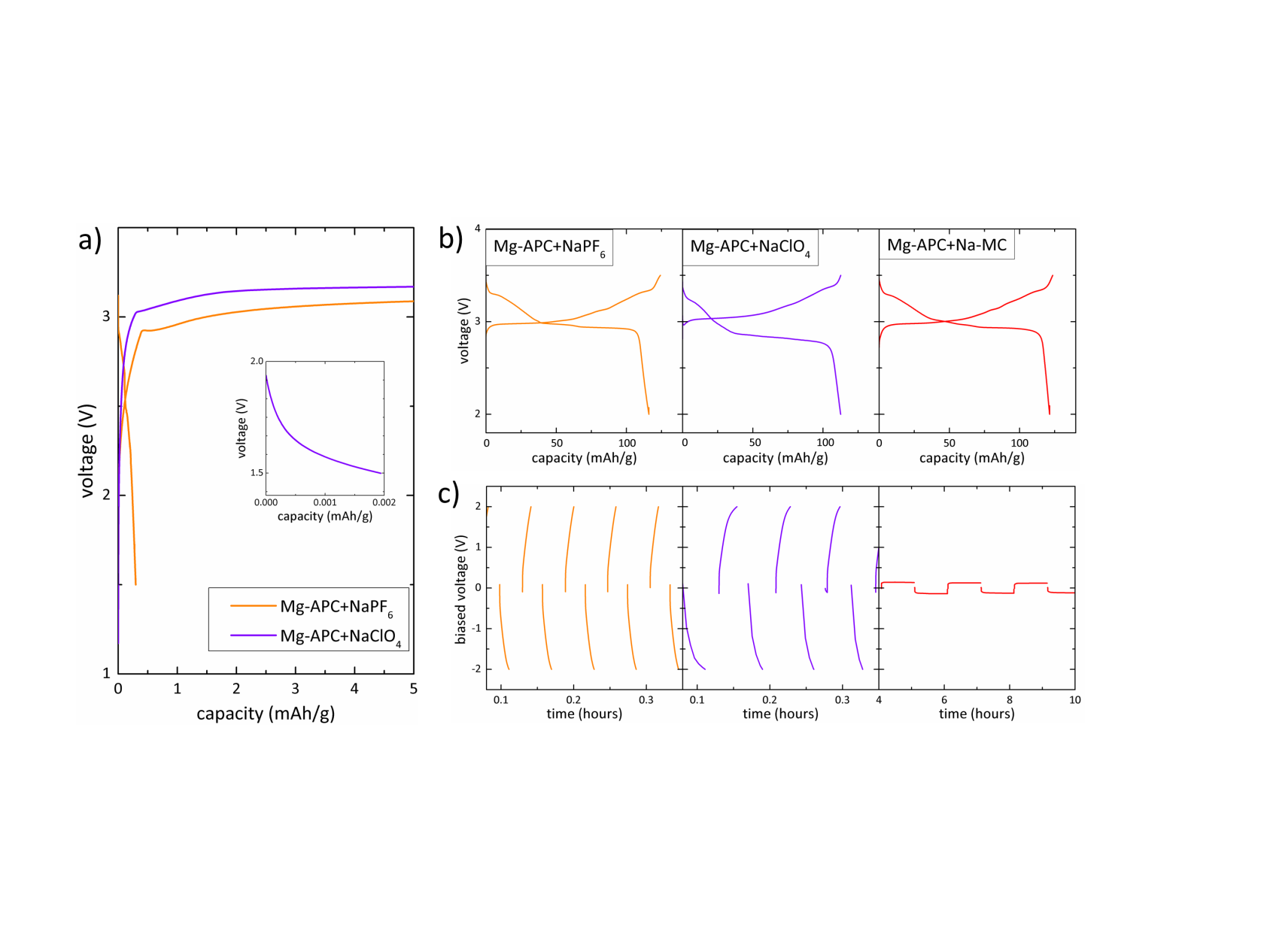


**Figure S2.** Evaluation of Na-intercalation and Mg deposition/dissolution in half cells. (a). Performance of Mg-NaCrO_2_ battery using different dual salt electrolytes. The insertion zooms in the data using Mg-APC and NaClO_4_ electrolyte. (b). Na-intercalation in different dual salt electrolytes. (c). Mg deposition/dissolution in different dual salt electrolytes.





**Figure S3.** Performance of desodiated Na_1-x_CrO_2_ as Mg cathode in APC electrolyte.


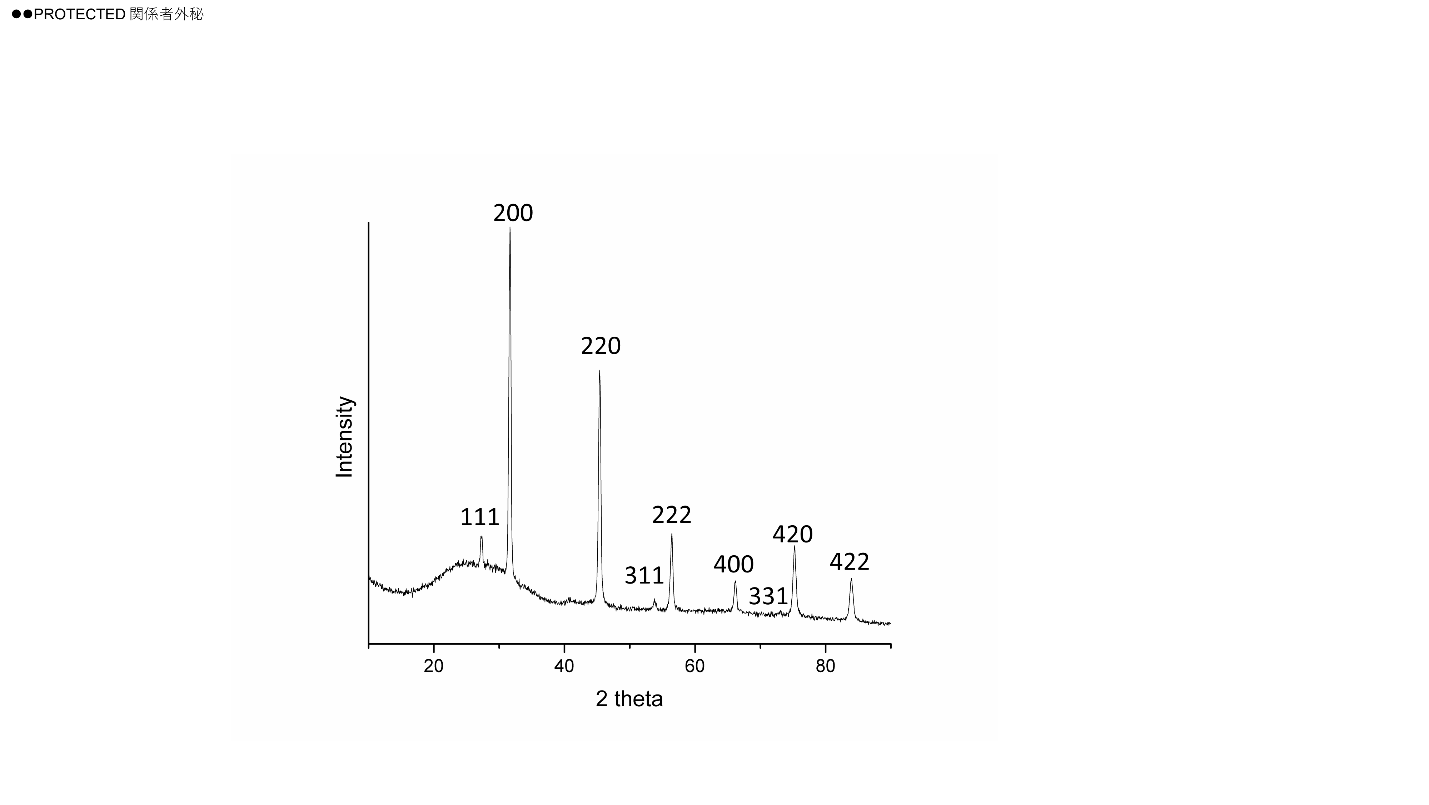


**Figure S4.** XRD of the precipitates of mixing Mg-APC and Na(CB_11_H_12_) electrolytes. All peaks are indexed to the diffraction of NaCl.
